# LPFG Biosensor for IL-6 Detection in Murine Serum Samples Associated with Ischemic Disease

**DOI:** 10.3390/s26092855

**Published:** 2026-05-02

**Authors:** Brenda Vertti-Cervantes, Karina González-León, Marcos García-Juárez, Georgina Beltrán-Pérez, Omar Montes-Narváez, Valentín López-Gayou, Oscar González-Flores, Hugo Martínez-Gutiérrez, Raúl Jacobo Delgado Macuil

**Affiliations:** 1 Instituto Politécnico Nacional, Centro de Investigación en Biotecnología Aplicada, Ex-Hacienda San Juan Molino Carretera Estatal Tecuexcomac, Tepetitla Km 1.5, Tlaxcala C.P. 90700, Mexico; bverttic2102@alumno.ipn.mx (B.V.-C.); vlopezg@ipn.mx (V.L.-G.); 2 Facultad de Ciencias Físico Matemáticas, Benemérita Universidad Autónoma de Puebla, Avenida San Claudio y 18 Sur, Col. San Manuel CU, Puebla C.P. 72570, Mexico; karina.gonzalezleon@viep.com.mx (K.G.-L.); georgina.beltranperez@viep.com.mx (G.B.-P.); 3 Centro de Investigación en Reproducción Animal UATx-CINVESTAV, Plaza Hidalgo Ote. 9, Cuarto Barrio, Panotla, Tlaxcala C.P. 90140, Mexico; mgarciaj@uatx.mx (M.G.-J.); 20209265@uatx.mx (O.M.-N.); ogonzalezf@uatx.mx (O.G.-F.); 4 Instituto Politécnico Nacional, Centro de Nanociencias y Micro y Nanotecnologías, Av. Luis Enrique Erro s/n, Nueva Industrial Vallejo, Gustavo A. Madero, Mexico City C.P. 07738, Mexico; humartinez@ipn.mx

**Keywords:** optical biosensor, interleukin 6 (IL-6), micro infrared spectroscopy, principal component analysis

## Abstract

**Highlights:**

**What are the main findings?**

- LPFG biosensor was developed using IL-6 mAb to detect interleukin 6 (IL-6).
- IR spectroscopy was used to determine functional groups associated with the presence of IL-6.

**What are the implications of the main findings?**

- Optical and electron micrographs demonstrated the morphological variations associated with each molecule on the surface of the optical fiber.
- Chemometric analysis was developed to correlate the experimental results.

**Abstract:**

Nowadays, optical fiber-based biosensors are widely used in various fields, particularly in medical diagnostics and the selection of appropriate treatments for certain diseases. One example is cerebral ischemic disease, where many biomarkers are released and provide valuable information about the severity and progression of the disease. In this study, a long-period fiber grating (LPFG) biosensor was developed using a standard single-mode optical fiber and monoclonal antibody (IL-6 mAb) as the biological recognition element to detect IL-6, which is a protein associated with the inflammatory process. The assembly of the LPFG biosensor was characterized through optical and electronic microscopy to observe morphological changes at different stages of fabrication and the detection process. Additionally, micro-infrared spectroscopy was employed to identify functional groups in the protein region linked to the presence of IL-6. Experimental data were analyzed using principal component analysis, confirming the biosensor’s ability to detect IL-6 and providing insights into its fabrication process.

## 1. Introduction

In recent years, several methods on optical fiber biosensors have been proposed to develop highly sensitive tools for clinical diagnostics and biosensing because these would allow the detection of real-time and in situ measurements, including surface plasmon resonance (SPR) [1,2,3,4], interferometric [5,6,7], optical ring resonator [8,9], photonic crystal [10,11,12] and tapered optical fiber [2,13,14,15]. Many research groups have explored modifying the surfaces of optical fibers to generate biosensors with different capabilities. In their work, Chen J. et al. (2023) [4] proposed a coreless multimode optic fiber-modified biosensor to detect L-glutamine; they fixed a gold film by chemical crosslinking, and through the same chemical reaction, the L-glutamine-binding protein (QBP) was attached onto the surface of the gold film. The authors mentioned that with their method, the optical biosensor had a sensitivity of 10.797 nm/log10 [G/n] in an in vitro embryo culture medium [4]. On the other hand, Makhsin SR. et al. (2023) developed a biosensor utilizing a single-mode optical fiber, in which a AuNP-agarose hydrogel film served as the sensing material to detect four glycerol concentrations [16]. Wang F. et al. (2023) developed an optic fiber tip biosensor to determine immunoglobulin G (IgG) using the human antibody IgG; via the microfabrication method, two-photon polymerization was printed in the optical fiber end face, propylene glycol monoethyl ether acetate (PGMEA) was added to finally yield a chromium film and a gold film was deposited by magnetron sputtering [17]. Wang R. et al. (2023) for their part utilized a metal–organic framework material fixed to a U-shaped optical fiber biosensor to detect the presence of mucin 1 in tumor tissues at concentrations as low as 1 pg/mL [3]. Moreover, Esfahani Monfared (2020) generated a review of the development of plasmonic optical fiber biosensors. This review showed how using different metals (gold, silver, nitrides, graphene, or transition oxides) makes it possible to obtain biosensors in different biology fields [18].

Among these techniques, long-period fiber gratings (LPFGs) stand out because of their high sensitivity. An LPFG is formed by a periodic modulation of the refractive index (RI) at the core of the optical fiber resulting from the coupling of the light between core and cladding modes [19]. Through their high sensitivity to surrounding refractive index (RI) changes, LPFGs represent a great technological platform that can be employed in a wide range of chemical and biological applications [20,21,22]. In 2024, Dyankov G et al. developed a double-resonance LPFG biosensor to determine the presence of Helicobacter pylori; they showed that LPFG reaches an accuracy of 10^2^ UFC/mL, whereas SPR reaches a sensitivity of 3000 UFC/mL [23].

IL-6 protein plays a critical role in several biological processes in humans. IL-6 is a protein with a molecular weight of 26 kDa, composed of 184 amino acids, two N-glycosylation sites, and four cysteine residues [24]. In healthy conditions IL-6 serum concentration level is under 4 pg/mL; however, in stress situations, disease response, or injury, the IL-6 level in serum can rise significantly, reaching several tens or hundreds of pg/mL [25]. Moreover, IL-6 is produced by different cells, including T and B cells, monocytes, macrophages, fibroblasts, endothelial cells, and tumor cells [26]. IL-6 exhibits pleiotropic effects on various physiological processes, including immune response, acute-phase reactions, and hematopoiesis. Its quick production contributes to host defense mechanisms during infections or tissue injuries [24]; IL-6 is also related to inflammatory conditions, tumor progression, hematological disorders [25], fibromyalgia [27], complications from chronic kidney diseases [28], and some viral diseases such as COVID-19 [29,30]. Furthermore, IL-6 increase has been correlated with acute respiratory distress syndrome, as well as with a greater need for mechanical ventilation, prolonged hospitalization, multiple organ dysfunction, and a higher likelihood of admission to intensive care units among patients with COVID-19 [31].

In this sense, the presence of IL-6 in plasma or tissues can serve as a valuable indicator for detecting certain diseases, particularly when correlated with the patient’s clinical history. Several methods have been employed to determine IL-6 levels, including immunoassays using serum and plasma samples from patients with conditions such as breast cancer. These immunoassays involve the indirect determination of the protein using an antibody bound to a colored substrate [32]. Currently, IL-6 measurements are commonly realized using commercial enzyme-linked immunosorbent assay (ELISA) kits, known for their high accuracy, precision, and sensitivity. However, the ELISA method requires long incubation times, substantial amounts of expensive reagents, and skilled technicians to execute multiple procedure steps, making automation a real challenge [33]. Thus, recognizing the importance of IL-6, the application of optical biosensors, developed on an optical fiber matrix, offers an alternative to traditional interleukin detection techniques.

Given the advancements, the objective of this study was to provide an alternative to conventional antibody-based detection methods by developing an optical device that enables detection of IL-6 expression associated with disorders that have global health implications. This involved generating an optical fiber biosensor for IL-6 protein detection in serum samples using self-assembled monolayers on the LPFG surface, inducing refractive index changes due to chemical and biological molecules added to the exposed area of the cladding, and observing the morphological changes associated with the molecule coupling. The self-assembly steps were meticulously characterized using micro infrared spectroscopy, optical microscopy, scanning electron microscopy, and chemometric analysis.

## 2. Materials and Methods

All chemical reagents were purchased from Sigma-Aldrich, (St. Louis, MO, USA) and were analytically reactive grade.

The optical fiber used for the assembly of the biosensors was Corning SMF-28e, with the specifications listed below: Cladding Diameter 125.0 ± 0.7 µm, Core Diameter 8.2 µm, Core-Clad concentricity ≤ 0.5 µm, Numerical Aperture 0.14, Zero Dispersion Slope (S_0_) 0.086 ps/(nm^2^·km) and Effective Group Index of Refraction (N_eff_) 1550 nm: 1.4682.

### 2.1. Long-Period Grating

The grating was engraved using two electrodes with the FITEL S176 fusion splicer (FITEL-S178A, Furukawa, Tokyo, Japan), utilizing a 65 mW electric arc coupled to an actuator that allows moving the optical fiber along the x-axis. The engraving of 20 points was performed with a periodicity of 620 µm, and the program used for the mechanical movement through the actuator was a Zaber console.

During the process, an optical spectrum analyzer (OSA 9057 F/8, Yokogawa, Tokyo, Japan) was used, with a CLD1015 laser diode (Thorlabs, Newton, NJ, USA) serving as light source to characterize the optical fiber behavior both at the beginning and at the end of the engraving. The optical fiber was coupled to the OSA using the fusion splicer. Characterization was carried out in the wavelength range from 1450 to 1250 nm.

### 2.2. Biosensor Assembly

This was performed using the monolayer self-assembly method, which was divided into five stages: recording a long period of grating over optical fibers, hydroxylation, functionalization, activation, and immobilization of the antibody used as a Biological Recognition Element (BRE).

For the assembly of the biosensor, single-mode SMF-28 optical fibers with a length of 80 cm were used as a support matrix. In the middle of the fiber, a 1.5 cm long polymeric cover was removed, and the recording of the LPFG was carried out using the point-point method. An electric arc of 65 mW was applied with a FITEL S176 fusion splicer, and 20 points were recorded with a period of 620 µm.

Hydroxylation was performed using potassium hydroxide (56 mg/mL) in a 1:1 solution of methanol and deionized water. At this stage, the optical fibers were placed in a Teflon plate container with the solution for 3.5 h in an extraction hood. Then, each optical fiber was rinsed with methanol and dried with nitrogen gas for 1 min.

Once the fibers were hydroxylated, they were functionalized for 1 h inside a glass coplin, staining with 200 µL of 3-Aminopropyltrimethoxysilane (3-APTMS) dissolved in toluene. Subsequently, the solution was removed, and the fibers were rinsed with toluene and dried for one minute with nitrogen gas.

The next stage was the activation, which was performed using 200 µL of a solution composed of N-hydroxysuccinimide (NHS) and 1-ethyl-3-(3-dimethylaminopropyl) carbodiimide (EDC) in phosphate-buffered saline (PBS). This solution was poured over the fiber and allowed to interact for 90 min with shaking at 200 rpm. Subsequently, the fibers were rinsed with PBS.

Finally, the antibody was immobilized under the same shaking conditions as in the previous stage. The IL-6 monoclonal antibody was deposited on the fiber by adding 200 µL of a PBS solution at a concentration of 0.002 µg/µL, with an interaction time of 2 h.

### 2.3. Interaction of the Biosensor Device

#### 2.3.1. Interaction with the Standard Proteins

For this stage, 200 µL of standard protein samples were used. The positive control (IL-6 standard) was at a concentration of 0.04 µg/µL, and the negative control (IL-10 standard) was at a concentration of 0.02 µg/µL. These controls were deposited on separate biosensors and stirred at 200 rpm for 2 h.

#### 2.3.2. Interaction with the Samples Obtained from the Ischemic Model

For the detection stage, blood serum samples were obtained from Sprague Dawley rats at 2 and 6 h post-ischemia. The animals were anesthetized with halothane (2% halothane in oxygen) and euthanized by decapitation. The blood sample was obtained from the trunk and centrifuged at 3000 rpm for 15 min to obtain serum, the serum was withdrawn, and the total volume was aliquoted (200 µL) and stored at −20 °C until processing. For detection, 200 μL of serum sample was placed in separate biosensors and kept under agitation at 200 rpm. Each biosensor was characterized by measuring the transmitted optical signal in real time with the optical spectrum analyzer over two hours at 5 min intervals.

### 2.4. Self-Assembly and Detection Stage Characterization

#### 2.4.1. Optical Microscopy

In each assembly stage, the surface morphology was observed using the microscope (Brand PME3, Olympus, Tokyo, Japan) with the 20× objective, and the images were saved using the MScopes application.

#### 2.4.2. µIR Spectroscopy

**Figure 1:**
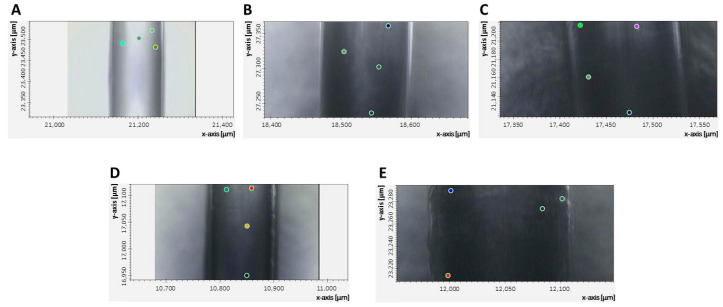
Optical micrographs using the Hyperion 15× by µIR spectroscopy in the region of 4000 to 400 cm^−1^ in reflection mode. (**A**) Optical fiber with a long-period grating (LPFG); (**B**) hydroxylation process; (**C**) functionalization process; (**D**) activation process; (**E**) immobilized antibody (biosensor).

The characterization was conducted using micro infrared (µIR) spectroscopy in reflection mode, for which a Hyperion microscope, coupled to a Vertex 70 infrared spectrometer, both from Bruker (Billerica, MA, USA), was used. Figure 1 shows the micrographs of the different stages of the self-assembly process. In each figure, some of the points chosen randomly for spectroscopic characterization were visible. The measurement region was the mid-infrared (4000 to 400 cm^−1^), and the spectra were the result of 32 scans at 12 different points across the entire biosensor area (1.5 cm in length). The self-assembly process, as well as the serum samples, were analyzed at several times. The experimental results were plotted using OriginPro 2025 software.

#### 2.4.3. Transmission Spectroscopy

To observe changes in the transmission spectrum, characterization was performed using an OSA 9057 F/8 optical spectrum analyzer (Yokogawa, Tokyo, Japan) with a super luminescent laser diode (CLD1015) emitting light in the wavelength range from 1250–1450 nm. The optical fiber was coupled using a FITEL-S178A splicer (Furukawa, Tokyo, Japan).

**Figure 2:**
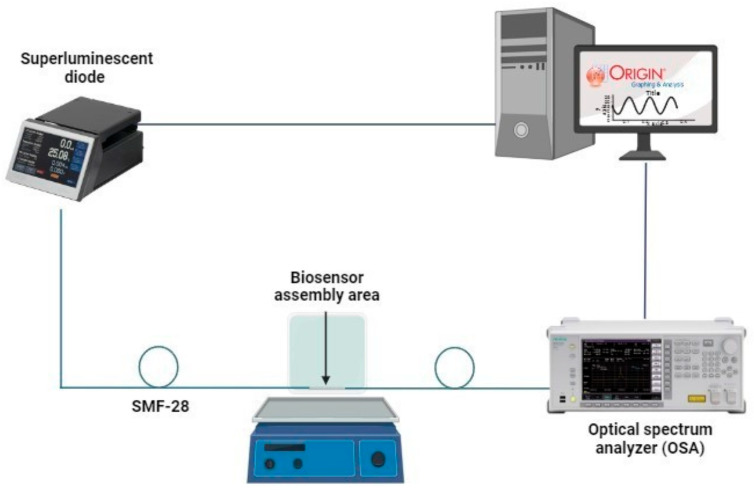
Set-up for transmission spectroscopy analysis.

The fibers with and without grid were characterized in each self-assembled stage: hydroxylation, functionalization, activation, immobilization, and detection. The characterization was carried out under the same agitation conditions; the experimental set-up is shown in Figure 2.

It is worth noting that this method was key to determining the suitability of each fiber before proceeding to the next assembly process. Changes in transmission spectroscopy were processed by PCA, and by observing the discrimination or clustering of these changes, it was decided whether each fiber would continue to the next stage.

### 2.5. Chemometric Analysis

Numerical analysis was performed using Principal Component Analysis (PCA), and the spectral regions with the most significant changes in the absorption signal were the ones chosen for the numerical analysis; the first region chosen was from 3750 to 3600 cm^−1^ (associated with amines I and II, and hydroxyls), the second region was from 3000 to 2800 cm^−1^ (associated with C-H bonds), the third region was from 1700 to 1250 cm^−1^ (associated with amide groups) and the fourth region was from 1000 to 600 cm^−1^ (associated with the skeletal molecular structure). PCA was performed using the OriginPro 2025 software.

### 2.6. IL-6 ELISA Analysis

Rat IL-6 ELISA kit (RAB0311) was supplied by Sigma-Aldrich (St. Louis, MO, USA). Rat IL-6 present in the samples binds to antibodies absorbed to the 96 microwells, ideal for serum, plasma, and cell culture with a sensitivity of 30 pg/mL, with a standard curve range from 40 to 100,000 pg/mL, measuring absorbance at 450 nm. The analysis was done for the 0- and 2-h samples.

## 3. Results

### 3.1. Characterization by Micro IR Spectroscopy

#### 3.1.1. Biosensor Assembly

**Figure 3:**
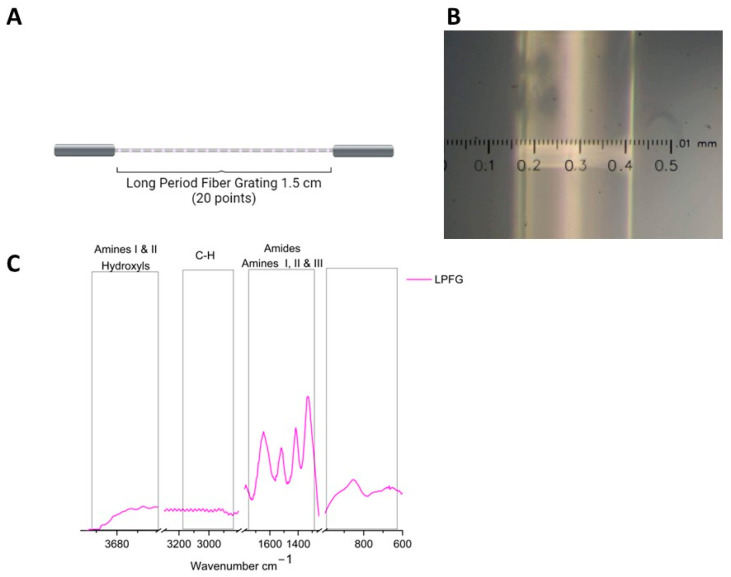
LPFG characterization. (**A**) shows the schematic representation of the LPFG recorded. (**B**) Optical microscopy of the LPFG recorded. (**C**) µIR spectra of the LPFG.

Once the LPFG was recorded on optical fiber, it was analyzed using an Olympus brand PME3 microscope with a 20× objective. Figure 3 shows the characterization of the LPFG, Figure 3A shows the schematic representation of the recording of 20 points made over a length of 1.5 cm with a period of 620 µm, Figure 3B shows the optical micrography in which the optical fiber surface and bright horizontal lines can be observed, attributed to the mechanical damage caused by the electric arc of 65 mW applied to the optical fiber and therefore to the LPFG generation. When performing characterization using µIR spectroscopy, the appearance of bands and peaks was observed. Figure 3C shows the infrared spectrum corresponding to the optical fiber in magenta, where the bands associated with Si-Si and Si-O bonds between 1000 and 600 cm^−1^ can be observed.

All experiments were done at room temperature, and to determinate the temperature influence in the spectra signals, for two hours we measured only the LPFG interacting with PBS at 26 °C with a humidity of 32%, where minimum spectral changes were observed with a 2.6 °C of variability (Figure S1).

**Figure 4:**
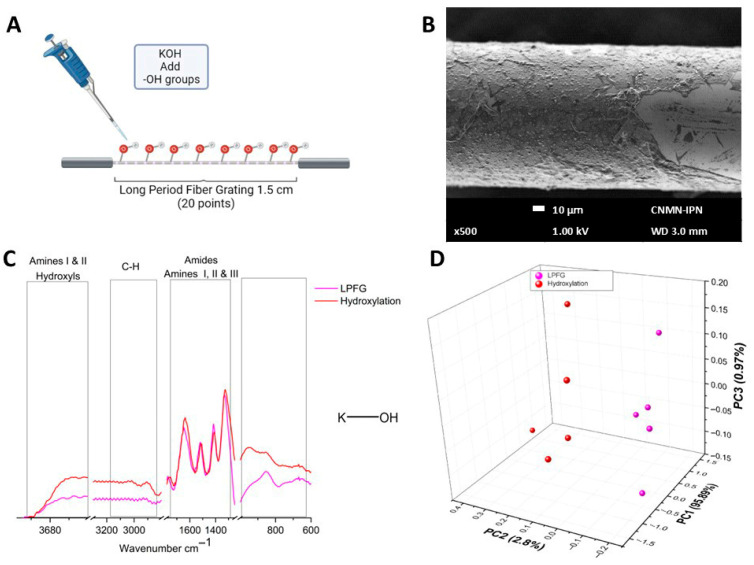
Hydroxylation stage characterization. (**A**) Schematic representation of the hydroxylation process. (**B**) SEM micrography of hydroxylated fiber. (**C**) µIR spectra of hydroxylation process. (**D**) PCA comparing LPFG vs. hydroxylation process.

Figure 4A shows a schematic representation of the assembly of hydroxyl functional groups on the surface of the optical fiber by adding potassium hydroxide. Figure 4B shows the SEM micrography image of the hydroxylated fiber, illustrating the morphological change resulting from the addition of potassium hydroxide and the formation of a layer on the optical fiber surface. In Figure 4C, the spectrum of the optical fiber after the hydroxylation process is shown in red, where the appearance of a peak at 954 cm^−1^ was observed, along with the attenuation of the peak at 851 cm^−1^ and the shift of the 1633 cm^−1^ peak. The most remarkable spectral changes were observed in the region from 3100 to 2800 cm^−1^, which was associated with C-H bonds and was chosen for principal component analysis. Figure 4D shows the results of the PCA analysis, in which PC1 accounts for 95.89% of the linear system, PC2 accounts for 2.8% of the linear system, and PC3 accounts for 0.97% of the linear system. As can be seen, the data corresponding to the LPFG (circles in magenta) have the tendency to cluster into negative values of PC2. In contrast, the cloud of points for the hydroxylation data (circles in red) clusters in positive values of PC2, associated with changes in the chemical structure of the optical fiber resulting from the addition of hydroxyl functional groups.

**Figure 5:**
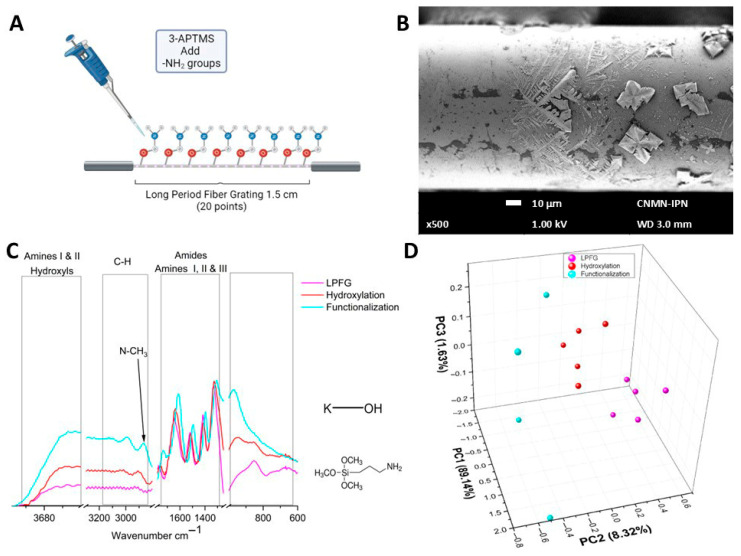
Functionalization stage characterization. (**A**) Schematic representation of the functionalization process. (**B**) SEM micrography of functionalized fiber. (**C**) µIR spectra of functionalization process. (**D**) PCA comparison of LPFG, hydroxylation, and functionalization processes.

Data from the functionalization procedure, which included the addition of 3-APTMS, is shown in a diagram (Figure 5A) depicting the incorporation of amino groups from the amino silane agent. The incorporation of 3-APTMS molecules causes changes in the surface shape of the optical fibers during the assembly processes, as seen in Figure 5B. The 500× SEM micrography shown exhibits morphological alterations, including fractal structures that form branches and spread over the surface of the LPFG. In Figure 5C the µIR spectrum of functionalization is shown in cyan. In this figure is is possible to observe the definition of a band at 965 cm^−1^, the shift of bands in the region of amides and amines towards lower frequencies, the appearance of a band at 2865 cm^−1^ corresponding to N-CH_3_ bonds associated with the incorporation of APTMS functional groups, and the definition of a band at 2983 cm^−1^. When performing the PCA in the region from 3000 to 2800 cm^−1^ (Figure 5D), it was observed that PC1 responded to 89.58% of the linear system, PC2 to 7.15%, and PC3 to 2.39% of the linear system, and that the LPFG data (circles in magenta) was now clustering in positive values of PC2, while the hydroxylation data (circles in red) remain at values close to zero. Finally, data clustering corresponding to the functionalization stage (circles in cyan) remain in negative values of PC2, indicating a greater difference in terms of chemical composition due to the incorporation of amine and amide functional groups onto the surface of the optical fiber.

**Figure 6:**
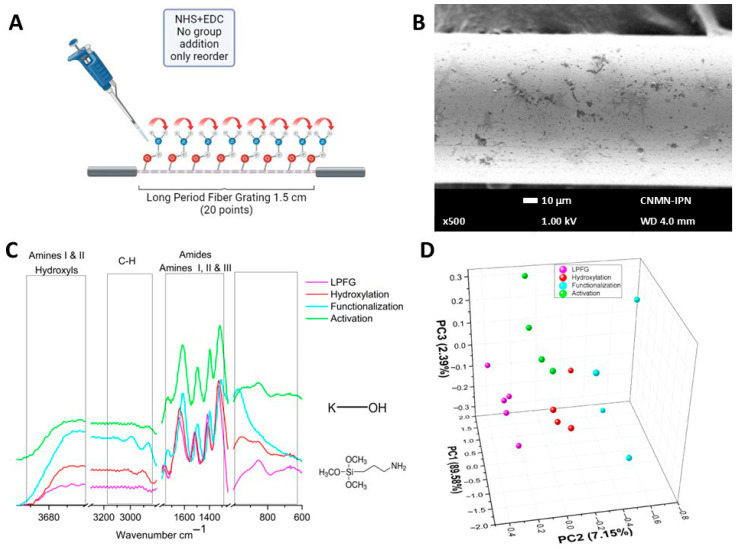
Activation characterization. (**A**) Schematic representation of the activation process. (**B**) SEM micrography of activated fiber. (**C**) µIR spectra of the activation process. (**D**) PCA comparison of LPFG, hydroxylation, functionalization, and activation processes.

Figure 6 shows the results obtained by characterizing the optical fibers after activation. As shown in the schematic representation of Figure 6A, this self-assembly step involves only a reorientation of existing functional groups, without the incorporation of new molecules, previously coupled with the objective that the biological recognition element can be linked correctly. As shown in Figure 6B (SEM micrography) the fractal structures disappeared, and there exists a uniform distribution of molecules on the optical fiber surface, which facilitate the incorporation of the antibody in the next stage. Figure 6C shows the µIR activation spectra in green, where greater homogeneity was observed in the bands due to the reorientation of the functional groups. Figure 6D shows the PCA performed on the data in the carbohydrate region, where PC1 explains 89.56% of the total variance of the linear system, PC2 accounts for 7.15%, and PC3 accounts for 2.39%. The data cloud of the LPFG remains in positive values of PC2. In contrast, the data from the following assembly stages were grouped in negative values of PC2.

**Figure 7:**
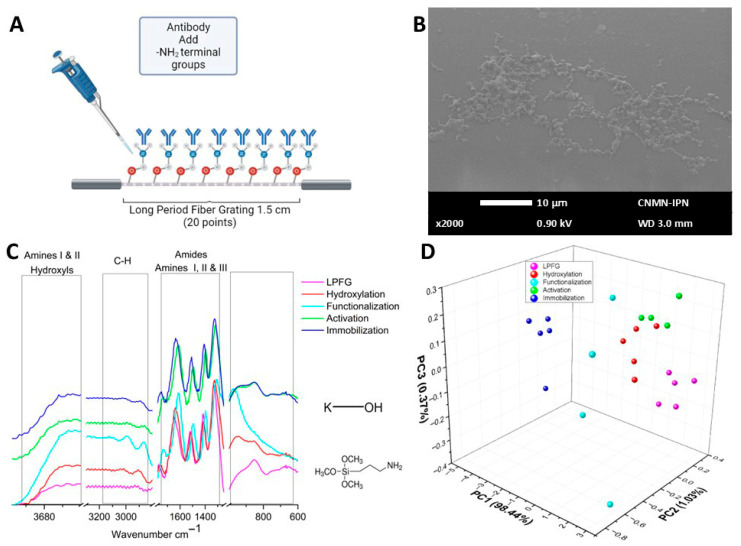
Antibody immobilization characterization. (**A**) Schematic representation of the immobilization process. (**B**) SEM micrography of the biosensor. (**C**) µIR spectra of immobilization process. (**D**) PCA of complete biosensor assembling.

Figure 7A schematically illustrates how the antibody was incorporated into the device, allowing the antibody light chains to recognize the protein of interest (IL-6). Figure 7B shows the SEM micrography in which the incorporation of antibodies to the surface of the biosensor device can be observed. Figure 7C shows the µIR spectral data of the immobilization process in blue. Although no apparent changes in banding were observed in Figure 7D, the PCA was done to compare the spectra obtained by the grouping data from each biosensor assembly stages; the first three principal components explain nearly 100% of variance of the linear system.

**Figure 8:**
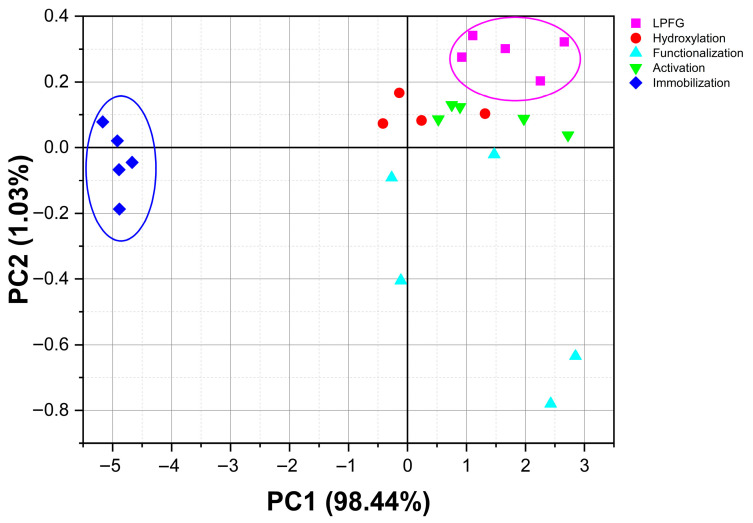
Comparison of µIR spectra behavior by the principal component analysis in the region from 3100 to 2800 cm^−1^.

Figure 8 show the principal component analysis for the region from 3100 to 2800 cm^−1^ corresponding to the C-H functional group region; it was possible to observe that the PC1 responds to 98.44% of variance of the linear system. Thus, as shown by the data clustering of LPFG in magenta and the immobilization stage of the antibody in blue, it was observed how they separated from the other stages, due to the compositional difference of molecules coupled on the surface of the optical fiber and their respective terminal functional groups, which could be associated with the biosensor development (raw infrared data in Table S1 and PCA data in Table S2).

**Figure 9:**
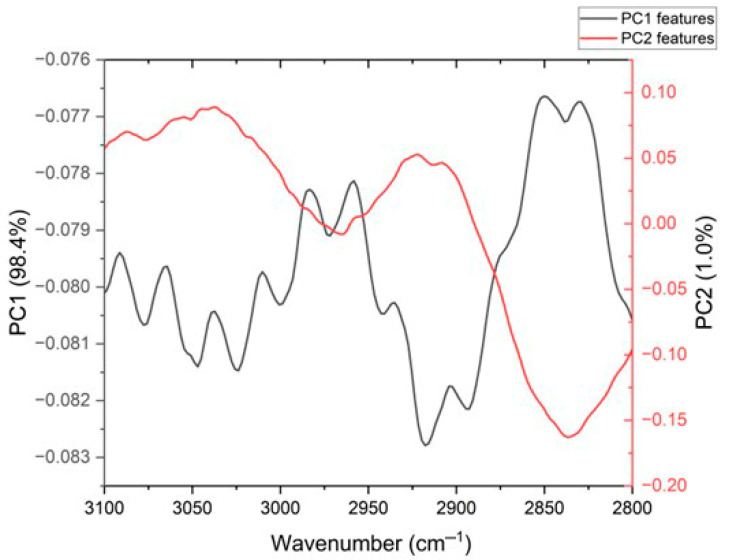
Spectral features corresponding to the first two principal components of the numerical analysis in the 3100–2800 cm^−1^ region.

In Figure 9 shown below, it is possible to observe the main principal component features that contribute to the percentage of variance. The dark line shows the features of PC1, with the main peaks at 2983 cm^−1^, 2958 cm^−1^, 2850 cm^−1^, and 2830 cm^−1^, while the red line shows the features of PC2, with the main peaks at 3086 cm^−1^, 3060 cm^−1^, 3036 cm^−1^, 2924 cm^−1^, and 2907 cm^−1^.

Table 1 below shows the wavenumbers corresponding to the spectral features of the first two principal components in the 3100–2800 cm^−1^ region for the biosensor self-assembling. It can be observed that these features were mainly related to the methoxy, methine, methyl ether (O-CH_3_), C-H, N-H, and amine functional groups.

#### 3.1.2. IL-6 and IL-10 Detection

**Figure 10:**
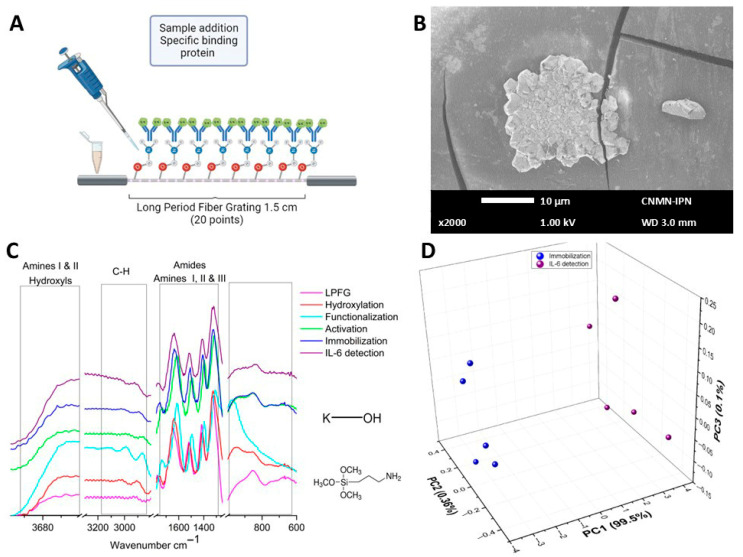
Detection process of positive control (IL-6). (**A**) Schematic representation of the positive detection process. (**B**) SEM micrography of IL-6 detection. (**C**) µIR spectra of the positive detection process. (**D**) PCA of antibody immobilization process vs. IL-6 detection.

Standard interleukins were used for the detection process. By interacting with the biosensor device with the positive control (IL-6), the data shown in Figure 10 were obtained. Figure 10A schematically represents the protein–antibody binding, and Figure 10B shows the SEM micrography of the biosensor surface, where conglomerate prismatic structures were observed, attributed to the binding of antibodies and antigens. Figure 10C shows the µIR spectrum of the detection process (purple line), revealing no apparent changes. PCA analysis was performed in the region of 3100–2800 cm^−1^, comparing the antibody immobilization process and detection stage (see Figure 10D). Thus, PC1 explained 99.5% of the linear system, PC2 explained 0.36%, and PC3 explained 0.1% of the linear system. As shown in the graph, PC1 effectively separates the experimental data corresponding to the detection, which was distinct from the clustering of the immobilization data, verifying the binding of the protein and resulting in a chemical modification of the biosensor structure.

**Figure 11:**
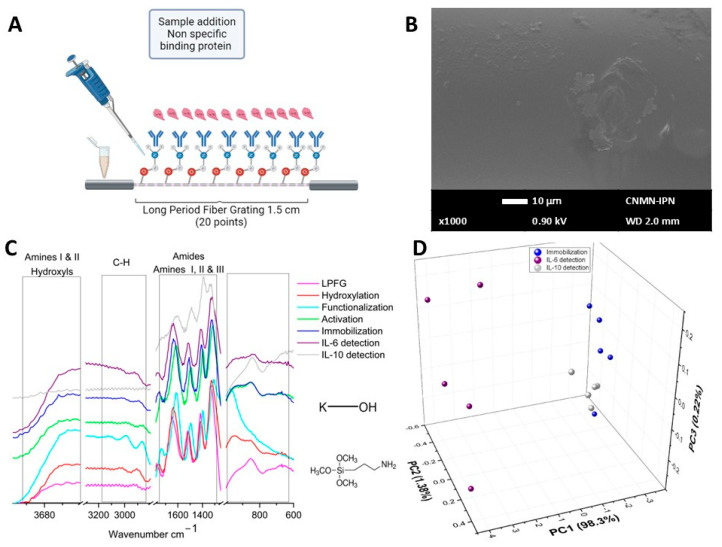
Detection process of negative control (IL-10). (**A**) Schematic representation of the negative detection process. (**B**) SEM micrography of IL-10 detection. (**C**) µIR spectra of the negative detection process. (**D**) PCA of antibody immobilization process vs. IL-6 detection vs. IL-10 detection.

The biosensor interaction with the negative control (IL-10) is illustrated in Figure 11. The schematic representation of this is provided in Figure 11A, where a non-specific protein (IL-10) and the biosensor generated (antibody IL-6) were expected not to interact. Observing the SEM micrography of Figure 11B, it was observed that the prismatic structures were no longer present and only some agglomerated amorphous structures in some areas of the biosensor were now present. Figure 11C shows the results of the µIR spectroscopy; the grey line corresponds to the interaction of the biosensor with the negative control. It was possible to observe evident spectral changes when comparing the data with the positive control, because the chemical structure of IL-10 differs from that of IL-6. Figure 11D shows the PCA for the antibody immobilization data compared with the detection data of the positive and negative controls. PC1 accounted for 98.3% of the linear system, PC2 for 1.38%, and PC3 for 0.22% of the linear system. The results show that it was possible to discriminate between the absence and presence of the analyte IL-6, since the data clustering of the positive control (IL-6) was maintained at positive values of PC1. In contrast, the data corresponding to the negative control (IL-10) clustered close to the spectral data of the biosensor device (with the specific antibody for IL-6), where both data groups remained at negative values of PC1. This means two things: that antibody IL-6 does not bind with IL-10 protein (used as negative control), and because of this, it’s possible that in the serum, where several proteins may be present, these biosensors were selective only for IL-6 interleukin, of course, if this protein was present in the samples.

### 3.2. Characterization by Transmission Spectroscopy

**Figure 12:**
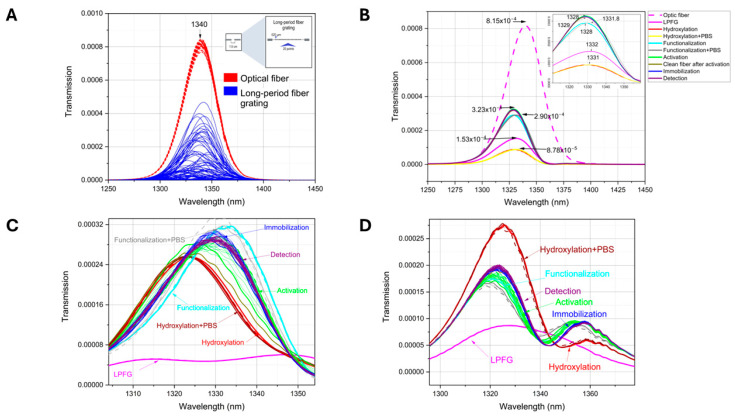
(**A**) Comparison of transmission spectra behavior before and after LPFG. (**B**) Transmission power attenuations were observed in the biosensor development and detection. (**C**) Assembly characterization and IL-6 detection. (**D**) Assembly characterization and IL-10 detection.

Each of the assembly stages and detections were characterized using transmission spectroscopy; Figure 12 shows the results of the assembly and interaction with the standard proteins. Figure 12A, in red, shows the spectrum corresponding to the optical fibers before the LPFG, and in blue the transmission spectra after etching. Each fiber exhibits distinct spectral behavior, indicating an affinity for specific wavelengths.

Figure 12B shows the representation of the results obtained in the optical spectral analyzer; in each stage the transmission power was variable due to the changes in the refractive index of the optical fiber, whose wavelength value was indicated with arrows; when zooming in on the region from 1310 to 1360 nm, it was possible to observe more clearly slight spectral shifts, which are indicated at the transmission peak with arrows as well as the wavelength value.

Figure 12C,D illustrate the results of transmittance response at different stages of the biosensor assembly process and detection. In the figures, magenta lines are the response associated with the LPFG into the optical fiber, the red lines are the response to the hydroxylation process, the cyan lines are associated with the functionalization process, activation in green lines, immobilization in blue lines, and finally in purple the IL-6 detection.

By observing the transmission spectral behavior, it was possible to discriminate the signals associated with LPFG and hydroxylation; in addition, the spectral behavior of both of these data (LPFG and hydroxylation) was perfectly discriminated between them and the rest of the stages. However, the spectra associated with the processes of detection, immobilization, functionalization, and activation showed minimal changes in the transmission intensity or very small spectral shifts. To try to obtain better discrimination, the experimental data were processed by principal component analysis method (raw data in Tables S3 and S5).

**Figure 13:**
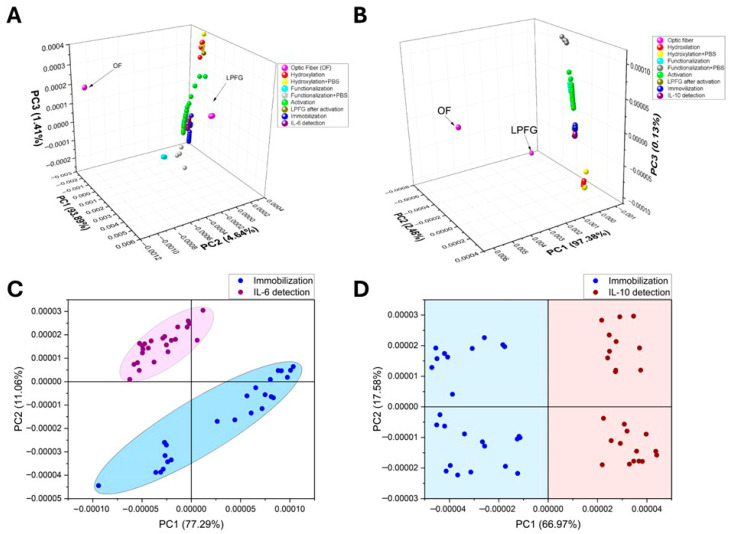
PCA from controls: (**A**) biosensor construction and detection to IL-6 (positive control); (**B**) biosensor construction and detection to IL-10 (negative control); (**C**) IL-6 detection vs. antibody immobilization; (**D**) IL-10 detection vs. antibody immobilization.

Figure 13 shows the PCA scores, graphing in red the data corresponding to hydroxylation, in maroon the data associated with hydroxylation plus the phosphate buffer solution, in cyan the data response associated with the functionalization process, in brown the data response of the clean activated optical fiber, in blue the response associated with immobilization and in purple the response corresponding to detection. It should be remembered that the activation, immobilization, and detection stages were followed in real-time, measuring every 5 min.

In both Figure 13A,B it is possible to observe the grouping clustering according to each stage of self-assembly and detection. This trend was most easily observed for the early stages of the biosensor assembly. For the data associated with the immobilization and detection stage, it is possible to observe how the data clustering moves towards positive values of PC3 as a function of the measurement time, which was associated with the number of molecules that bind to the modified surface of the optical fiber (antibody immobilization) or the number of interleukin molecules that were recognized by the biosensor device (detection).

Once it was possible to discriminate between the different stages of the biosensor device generation process, Figure 13C,D present the principal component analysis performed for the immobilization and detection data of two assembled biosensors. In Figure 13C by performing numerical analysis only of the results of these two stages, it can be seen how the data of the immobilization stage have the tendency to group diagonally, distributed along PC1, held within the confidence ellipses. With this, it was possible to completely discriminate between the absence (immobilization) and presence (detection) of IL-6 in the analyzed plasma samples. On the other hand, Figure 13D shows the results of immobilization and detection of IL-10 (negative control), where it was possible to observe how the data were distributed completely differently throughout PC2 and were discriminated against in PC1 (raw data in Tables S4 and S6).

**Figure 14:**
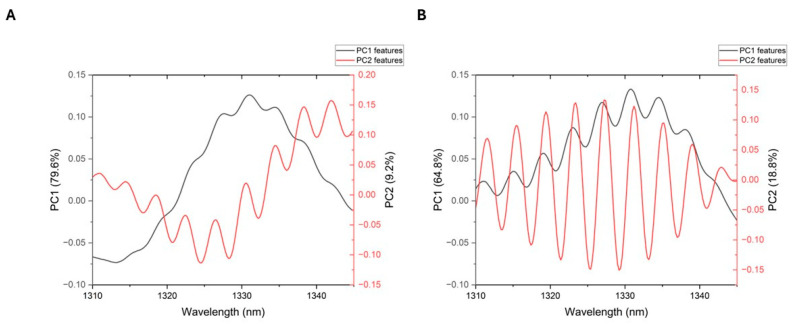
Spectral features corresponding to the first two principal components of the analysis in the 1310 to 1345 nm region. (**A**) PCA features of the positive control and (**B**) PCA features of the negative control.

The graphs below show the spectral features resulting from PCA for the positive control (Figure 14A) and the negative control (Figure 14B). In the figures, the dark line shows the PC1 features, and the red line shows the features related to PC2, which contribute to the percentage of variance in the numerical analysis.

Table 2 summarizes the locations of the nine bands associated with these features, as well as their corresponding wavelength (nm) and wavenumbers (cm^−1^), associated with the spectral characteristics of the C–H stretching and C–H deformation functional groups of carbohydrates.

**Figure 15:**
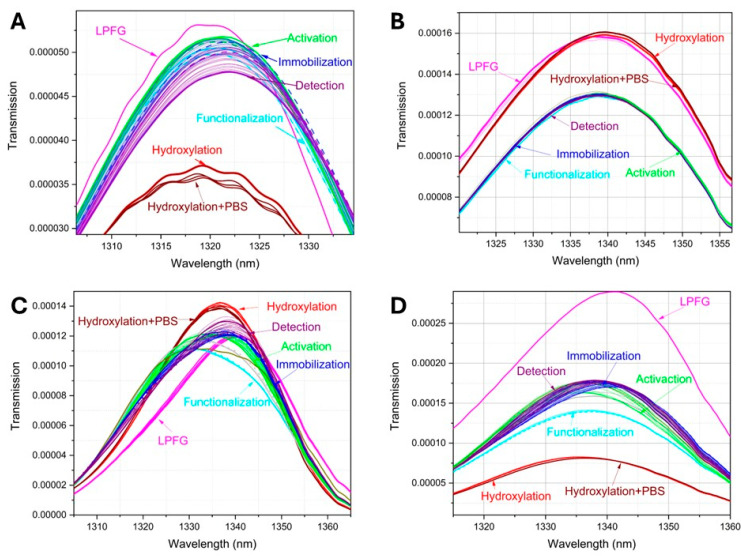
Transmission spectra of the assembled biosensors for IL-6 detection. (**A**) Biosensor used for detection in the 2 h sample without treatment. (**B**) Biosensor used for detection in the 2 h sample with estradiol benzoate. (**C**) Biosensor used for detection in the 6 h sample without treatment. (**D**) Biosensor used for detection in the 6 h sample with estradiol benzoate (raw data in Table S7).

In Figure 15, the transmission spectra behavior corresponding to the biosensors used for detection at 2 h and 6 h samples, with and without estradiol benzoate treatment, are shown. Upon observing the transmission signals it was possible to determine that for the signals associated with LPFG and hydroxylation, the transmission behavior was perfectly discriminated between them and the rest of the stages. However, the spectra associated with the processes of detection, immobilization, functionalization, and activation show minimal changes in power intensity, as well as slight spectral shifts. In fact, it was also possible to observe how the PBS solution does not generate significant changes in the optical response; see also Figure S1.

**Figure 16:**
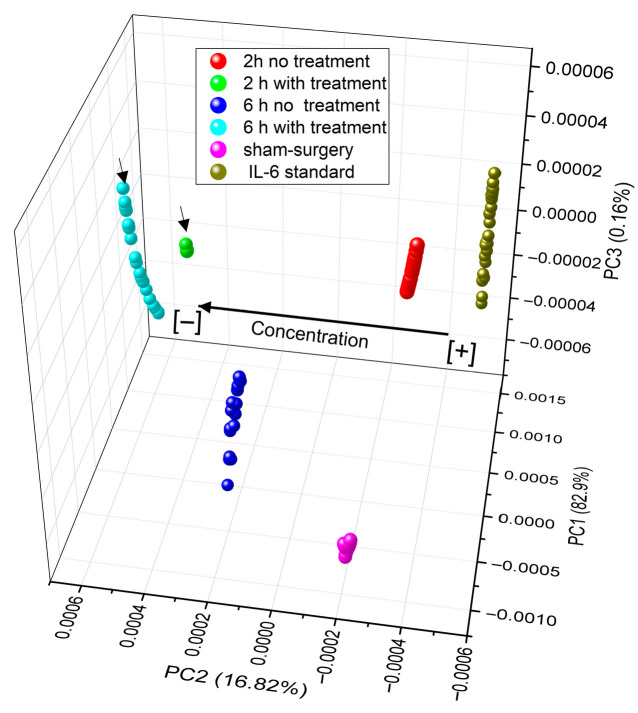
Principal component analysis comparing the transmission data from the detection stage in samples without and with estradiol benzoate treatment at two induction times (2 and 6 h), as well as the controls: positive (IL-6) and negative (IL-10).

To attempt to achieve better discrimination, Figure 16 shows the numerical analysis for the experimental data for the detection stage using the principal component method; data of the positive control IL-6 [0.04 µg/µL] are shown in olive green, in magenta data of the Sham-surgery control sample, in red data of the 2 h untreated sample, in green data of the 2 h sample with estradiol benzoate treatment, in blue data of the 6 h untreated sample, and in cyan data of the 6 h sample with estradiol benzoate treatment (raw data in Table S8).

In the analysis presented above, it is possible to observe clustering associated to each cloud of points based on the type of detection performed. The data points in cyan and light green, associated with samples treated with estradiol benzoate (which are marked with arrows), cluster in positive values of PC2; the same behavior was observed in the data cloud of the biosensor interacting with the sample at 6 h without treatment, which is also found in positive values of PC2. The data cloud corresponding to the positive control (IL-6 standard) clusters in the negative values of PC2 and positive values of PC1. The samples associated with the negative control (Sham-surgery) also tend to cluster in negative values of PC2 and PC1, indicating that the developed biosensor can discriminate between the absence of IL-6 and the standard of this protein. A curious fact is that the data from the biosensor interacting with the untreated sample 2 h after the induction of the ischemic process tend to cluster near the positive control data, which would indicate that at this time there is a higher concentration of IL-6 in the rats induced to the ischemic process.

Therefore, the principal component analysis shows that samples with lower IL-6 concentration were found in positive values of PC2, while samples with higher IL-6 concentration tend to cluster in negative values of PC2.

### 3.3. ELISA Results

To correlate the spectroscopic techniques, ELISA analysis was done for samples of 0 and 2 h of the ischemic model; the results show that the concentration was 11.5 and 6 pg/mL, respectively, and the microwell image is shown in Figure S2.

Table 3 presents a comparison of studies conducted between 2024 and 2026 focused on the biodetection of IL-6 [34,35,36,37]. This table shows the characteristics of each study, including detection method, sample characteristics, response time, limit of detection (LOD), and a brief description of its operation. It is evident that the detection of this protein remains of clinical interest and the opportunity and limitation areas.

## 4. Discussion

Optical biosensors have great development in all scientific fields. Matrix modification to bond molecules into the surface is a crucial role before adding the Biological Recognition Element (BRE). The optical biosensors based on optical fibers have a great demand in several chemical and biochemical applications. These devices had the principle on monitoring the surrounding refractive index changes induced by the BRE and the analyte interaction. In our study an LPFG was recording on the optical fiber surface, with 20 points made over a length of 1.5 cm with a period of 620 µm. A review of Jintao, et. al. [38] mentioned that several kinds of interferometers or resonances can be developed, with or without cladding removal, with a period interval from 100 to 1000 mm, in transmission or reflection mode. Figure 3B shows the mechanical damage generated in the core of the optical fiber, with a period within the range reported by other authors.

Armistead, et al. [39] reported how the hydroxylation stage generates two distinct types of hydroxyl sites which allow or ensure better binding sites than only the Si-Si or Si-O functional groups of the SiO_2_ matrix. In Figure 4, the appearance of new bands can be observed; the biggest spectral changes were observed in the region of the C-H bonds. Figure 4D shows the PCA results where it was possible to observe that the data corresponding to the LPFG were grouped into negative values of PC2, whereas the cloud of points of the hydroxylation data were grouped in positive values of PC2 associated to refractive index modification in the chemical structure of the optical fiber by the addition of hydroxyl functional groups in this stage.

Data from the functionalization stage, which included the addition of 3-APTMS, causes the shape of the optical fiber surface to change with the following assembly processes, as seen in Figure 5B; the SEM micrograph shows the surface modification in the LPFG by the addition of this molecule where it was possible to observe fractal structures that formed branches and spread over the LPFG surface. The infrared spectral changes in intensity were associated with a covalent bond between the 3-APTMS and the OH, Si-Si, and Si-O bonds presented in the optical fiber surface. Similar results were obtained by Chen C-F. et al. (2006) applied APTMS to immobilize molecular recognition elements through either covalent coupling or electrostatic interactions [40]. Nakagaki et al. modified the silica matrix with 3-APTMS with the intent to bond amino groups on the solid, and in this way generate amino-propyl silica (APS) particles in the matrix surface [41], via a peptide that binds the APS to the carbonyl chloride on the metalloporphyrin. Moreover, Sandor M. et al. in 2016, using aminopropyl-functionalized silica nanoparticles, showed that the mainly infrared bands between 3800 and 3000 cm^−1^ were associated with Si-OH groups, the bands between 3000 and2800 cm^−1^ to the CH_2_ groups, the band centered at 1630 cm^−1^ characteristic for the NH_2_ group, the band centered at 1650 cm^−1^ assigned to double CO bond in stretching vibration mode, the peak at 1491 cm^−1^ for the N-H in the bending vibration mode associated to the primary amine group (R-NH_2_), and finally the band at 1390 cm^−1^ was assigned to the double CO in the bending vibration mode [42]. Figure 5C displays similar spectral behavior except the band centered at 1650 cm^−1^, which was absent from our samples. The PCA results for the functionalized process clearly show that the experimental data clustering in the region from 3000 to 2800 cm^−1^ (as shown in Figure 5D) has well-identified groups in terms of the chemical composition, based on the incorporation of amine and amide groups to the surface of the optical fiber. Similar results were obtained by Casarino A. F., et al. in 2021 but using a multivariate curve resolution model applied to their FTIR experimental results [43].

Sam S. et al. in 2010 showed how chains of carboxyl-terminal (like the 3-APTMS) by chemical reaction with EDC-NHS generate as pure as possible succinimidyl ester-terminal surfaces [44]. In our FTIR results (see Figure 6C) a reorientation of the groups was performed, which could be observed as a shift to higher frequencies. In addition, the PCA results for this stage still show good clustering according to each biosensor stage.

Some of the main metabolites to determine in biological samples are proteins, which are molecules with several functions within a cell; therefore, rapid, low-cost detection of proteins is crucial in any research field. Lyu S. et al. 2022 generated a review of the main optic fiber biosensor development to determine proteins in several samples [20]; this review mentioned how the surface of the optical fiber with different modifications can increase the binding capacity of the bioreceptor or biological recognition element. Nevertheless, these works emphasized improving the biosensor signal or sensitivity, ensuring that the functional modification was a crucial process. However, none of these works developed a characterized protocol to analyze step by step the biosensor assembling. Additionally, via chemometric analysis and electronic or optical micrographics, they did not correlate how molecules added to the optical fiber surface modify the optical signal; very few use a long period fiber grating to enhance the sensitivity of the optical fiber biosensor. In another review, Leung A. et al. (2007) mentioned several works that design optical fiber biosensors by tape of surface plasmon resonance [45]; in this review a very complete analysis of the theoretical or numerical theory was immersed in this kind of device; one of them is the evanescent wave. In our case, Figure 7A illustrates the mechanism by which the antibody was integrated into the device, allowing the antibody light chains to remain unobstructed and for us to be able to recognize the protein of interest. Notably, the optical microscopy images presented in Figure 7B reveal a rough surface topography, which can be attributed to the incorporation of the antibody. Spectral data from the immobilization process is presented in Figure 7C, while the numerical analysis in Figure 7D demonstrates the clustering of the data from each biosensor assembly stage. Importantly, the first three principal components of this analysis successfully resolved nearly 100% of the linear system. Since the molecules attached to the optical fiber’s surface and their corresponding terminal functional groups differ, it was possible to observe how the data from each stage of the biosensor construction process were entirely discriminated in Figure 8, which depicts the 2D PCA analysis of the whole process. Yuan S. et al. 2026 develop a D-Shaped Optical Fiber Sensor, enhancing their sensitivity by controlling the layer thickness with the aim to achieve optimal electric field confinement, showing that the layer thickness can generate effects in the optical signal response; in our case the self-assembled monolayer protocol shows a good reproducibility, which can be observe in the PCA figures; no matter which stage or biosensor data was analyzed, the PCA clustering shows differences between each biosensor stage or biosensor determination (interacting with controls or serum samples) [46].

Finally, the sensing ability was determined by the Interleukin 6 standard and the discrimination capacity against interleukin 10 as the negative control (Figure 10 and Figure 11, respectively). The optical micrographs demonstrate noticeable differences in biosensor morphology; it was observed that the LPFG had a greater amount of sample adhered to its surface. These changes were clearer with SEM; the SEM micrographs illustrate how the proteins were on the surface of the optical fiber, where the formation of prismatic structures can be observed. Meanwhile, Figure 11B presents a morphology very similar to that of the immobilization stage, with some amorphous structures attached to the surface. The behavior of the micro infrared spectra showed significant differences between the detection of IL-6 and IL-10 (Figure 10C and Figure 11C, respectively). When PCA was applied to the experimental results, the bands present in both detections were very different; the clustering showed good discrimination between IL-6 detection by the optical biosensor device (see Figure 10D); in contrast, when the optical biosensor interacted with the negative control (IL-10), the grouping of data was quite like the immobilization stage (see Figure 11D). This indicates the high selectivity provided by the optical fiber biosensor in addition to the fact that the determination of IL-6 was performed in less than one hour.

The results obtained from our study can be compared with those of other IL-6 detection methods reported in the literature. For instance, Huang et al. (2020) established a new IL-6 kit based on an immunofluorescent assay boast, which has a wide linear range (2–500 pg/mL) and good sensitivity (0.37 pg/mL); however, it is still a molecular method [47]. Similarly, Rahbar M. et al. (2021) developed a lateral flow assay via IL-6 colorimetric detection in human serum samples, offering a detection range within physiological levels (5 to 1000 pg/mL) [48].

In our study, selectivity was the focus of the investigation, and the subsequent stage will involve quantification and potentially enhancing the sensitivity of the biosensor. By refining the biosensor’s sensitivity, we aim to achieve more accurate and precise detection of IL-6 levels in serum samples, thereby contributing to advancements in biomedical research and clinical diagnostics.

Transmission results show a good correlation with the micro infrared results; the transmission spectrum shows changes according to the different molecules binding to the optical fiber surface; our results agree with the review analysis done by Cosimo Trono in 2024, where it was shown how different contributions increase the LPFG sensitivity, with a high level of technological readiness [21].

The PCA analysis of the transmission data shows again the good clustering in function of the optical biosensor development and during the interleukin determination (used as positive and negative controls); in fact the spectral features of the two first PC were related to the C-H functional groups, associated to carbohydrates, which agrees with infrared spectroscopy observations. Even the transmission results alone could be insufficient for interleukin determination; PCA can help us to enhance the differences between which molecules were present in the LPFG surface. Shackart KE and Yoon JY in 2021 showed how the use of multivariate methods can help to improve the results during the specificity and selectivity of the biosensor devices [49].

Spectral behavior shows differences according to each self-assembly step [50], and also for each biosensor detection in function of each different sample. When PCA is applied to spectroscopic data it improves these differences; the above demonstrates that the self-assembled and biosensor detection processes are reproducible.

Finally, Figure 13 shows how the 2 h experimental data cluster close to IL-6 standard, whereas the samples of 6 h without treatment and 2 and 6 h with treatment cluster far away the IL-6 PCA data points, where the PC2 positive values are associated to lower IL-6 concentration. This results in correlation with ELISA results, meaning that with the use of LPFG it could be possible to reduce the LOD lower to 6 pg/mL. Wang F. et al. in 2025 showed how, with a fiber Bragg grating enzymatic biosensor, they could reach a wide measurement range (10^−8^ to 10^−2^ M), similar to the sensitivity obtained in the present work [51]. In fact, the sensitivity obtained for IL-6 in the present work was higher than that obtained by Cai J., et al., in 2025 using a small period LPFG for glucose (1.95 nM); however, Cai et al. showed that using Bragg reflection exhibited a thermal sensitivity of 10 pm/°C, making LPFG biosensors a convenient and efficient matrix to measure multiple parameters in a simultaneous way [52].

## 5. Conclusions

This work conducted a comprehensive study and a detailed analysis of a robust long-period fiber grating biosensor capable of determining the presence of IL-6 in serum samples, where several proteins could be present, comparing IL-6 standard detection vs. IL-10 standard detection, as positive and negative controls. The PCA characterization, using infrared and transmission results, shows the compositional difference of molecules coupled on the surface of the LPFG biosensor. Also, PCA results show, for IL-6 standard detection, that PC1 completely separates IL-6 detection from the immobilization and negative control data, verifying that there exists a binding only with the protein of interest. Moreover, the optical and electron micrographs definitively demonstrated the distinct morphological variations associated with each molecule on the surface of the optical fiber. The scanning electron microscopy (SEM) images were the most effective in defining the structural changes generated after each stage of the self-assembly process and detection.

Unlike conventional techniques, this device is easy to use, requires no specialized personnel, and sample pretreatment is simple, requiring only centrifugation. Furthermore, the spectral data does not need to be preprocessed before principal component analysis (PCA), making this type of biosensor a potentially useful tool for future clinical diagnostics.

Something important to highlight is that, as far as we know, there are no works which developed a characterized protocol to determine the structural changes, associated to refractive index changes, according to the assembly of the biosensor device stage by stage, also showing through a chemometric and electronic or optical micrographic analysis, to correlate how the molecules were added to the surface of the LPFG. In addition to that, only in very few cases was a long period fiber grating used to improve the sensitivity of the optical fiber biosensor, as was demonstrated by ELISA result correlation, where a LOD below 6 pg/mL was obtained with biosensing samples at 2 h post-ischemia with our optical device.

## Figures and Tables

**Table 1:** Features for the biosensor self-assembly and the associated bands to the first two principal components; analysis was done in the 3100–2800 cm^−1^ region.

PC1 (98.44%)		PC2 (1.03%)	
Wavenumber (cm^−1^)	Spectral Feature Related	Wavenumber (cm^−1^)	Spectral Feature Related
2830	Methoxy, methyl ether O-CH_3_, C-H stretch		
2850	Methylamino, N-CH_3_, C-H stretch		
2903	Methine C-H stretch	2907 2924	Methine C-H stretch
2935	Methylene C-H asym/sym stretch		
2958	-CH3		
2983 3010 3037 3064 3090	N–H stretch, amines	3036 3060 3086	N–H stretch, amines

**Table 2:** Associated bands with spectral features to the first two principal components of the analysis in the transmission spectra behavior.

Positive Control				Negative Control				Spectral Features Related
PC1		PC2		PC1		PC2		
nm	cm^−1^	nm	cm^−1^	nm	cm^−1^	nm	cm^−1^	
1310.23	7632.25	1310.99	7627.77	1311	7627.76	1311.52	7624.74	C-H stretching + C-H deformation (carbohydrates)
1315.19	7603.46	1314.51	7607.40	1315.06	7604.22	1315.47	7601.85	
1319.32	7579.66	1318.50	7584.38	1319.11	7580.87	1319.39	7579.26	
1323.52	7555.61	1322.38	7562.12	1323.01	7558.52	1323.38	7556.41	
1327.58	7532.50	1326.47	7538.81	1326.96	7536.02	1327.30	7534.09	
1330.95	7513.43	1330.60	7515.41	1330.81	7514.22	1331.23	7511.85	
1334.59	7492.94	1334.46	7493.67	1334.46	7493.67	1335.14	7489.85	
1338.34	7471.94	1338.28	7472.28	1338.11	7473.23	1339.13	7467.53	
1342.30	7449.90	1341.96	7451.79	1341.89	7452.18	1342.95	7446.29	

**Table 3:** Comparison of studies conducted between 2024 and 2026 focused on the biodetection of IL-6.

Biosensor	Detection Method	Type of Detection	Sample Used	Sample Quantity Required	Incubation Time	Operating Principle	LOD	Reference
Label-Free Electrochemical Interleukin-6 Sensor Exploiting rGO-Ti_3_C_2_T_x_ MXene Nanocomposites.	Electrochemical.	Quantitative	Commercial human AB serum with standard protein was used	5 µL	16 min	Antibody-functionalized nanocomposite platform on a screen-printed nanochip.	2.1 pg/mL	[34]
MIP-Modified Porous Silicon Optical Sensor for Interleukin-6 Label-Free Quantification.	Optical in Psi matrix.	Quantitative	The standard protein in PBS was used	200 µL	1 h	Electrochemical etching on porous silicon followed by electrodeposition of fine molecular polymer.	300 ng/mL	[35]
Interleukin-6 electrochemical sensor using poly(*o*-phenylenediamine)-based molecularly imprinted polymer.	Electrochemical.	Quantitative	Standard solution in PBS.	-	15 min	Molecularly imprinted polymer on an AuNP-functionalized carbon electrode.	1.74 pg/mL	[36]
3D RGO@MoS_2_/Ag NPs based SERS platform for label-free detection of IL-6.	Optical substrate for Raman scattering	Quantitative	Standard solution in PBS	500 µL	1 h	SERS substrate functionalized with AgNPs and analysis by Raman scattering.	0.41 pg/mL	[37]
This work.	Optical on cylindrical matrix of LPFG.	Semi- Quantitative	Standard proteins in PBS and ischemic rat serum samples.	200 µL	1 h	Construction using self-assembled monolayers and detection based on spectral changes.	<6 pg/mL	

## Data Availability

The data sets generated and analyzed during the current study are available from the corresponding author upon reasonable request.

## Supplementary Materials

The following supporting information can be downloaded at: https://www.mdpi.com/article/10.3390/s26092855/s1, Figure S1: Temperature influence; Table S1: Raw data related to Figure 8; Table S2: PCA data related to Figure 8; Table S3: Raw data related to Figure 12C; Table S4: PCA data related to Figure 13A; Table S5: Raw data related to Figure 12D; Table S6: PCA data associated to Figure 13B; Table S7: Raw data related to Figure 16; Table S8: PCA data associated to Figure 16; Figure S2: Plate of ELISA test.

## Abbreviations

The following abbreviations are used in this manuscript:

LPFG	Long Period Fiber Gratings
LPG	Long Period Gratings
OSA	Optical Spectrum Analyzer
BRE	Biological Recognition Element
µIR	Micro Infrared
FTIR	Fourier Transform Infrared
IL-6	Interleukin 6
IL-10	Interleukin 10
PC	Principal component
OF	Optic Fiber
