# Supplementary material for: LPFG Biosensor for IL-6 Detection in Murine Serum Samples Associated with Ischemic Disease
Source: Sensors (Basel). 2026 May 2;26(9):2855. doi: 10.3390/s26092855 (PMC13165805; doi:10.3390/s26092855)
Supplement: Supplementary file 1 [file sensors-26-02855-s001.zip › Figure S1.pdf]

LPFG biosensor for IL-6 detection in murine serum samples associated with ischemic disease.

Table. Temperature influence experiment data obtained.

Measurem with PBS						
**Date**	02/05/2024	**Start time**	11:20 a. m.	**Environmental conditions**	**ºT**	26ºC
**ºT OF MZI**	26.3 ºC				**Humidity**	32%

**Measurement time**	**ºT**	**Environmental conditions**	
		**ºT**	**Humidity**
0 min	22.4 ºC	26 ºC	32%
5 min	23.8 ºC		
10 min	23.8 ºC		31%
15 min	23.9 ºC	27ºC	
20 min	24.1 ºC		30%
25 min	24 ºC		
30 min	24.1 ºC		
35 min	24.2 ºC		32%
40 min	24.2 ºC		
45 min	24.2 ºC		
50 min	24.2 ºC		31%
55 min	24.3 ºC		
60 min	24.2 ºC		
65 min	24.5 ºC	28 ºC	30%
70 min	24.4 ºC		
75 min	24.6 ºC		
80 min	24.5 ºC		29%
85 min	24.6 ºC		
90 min	24.6 ºC		
95 min	24.7 ºC		27%
100 min	24.6 ºC		
105 min	24.9 ºC		
110 min	24.8 ºC	29 ºC	29%
115 min	24.9 ºC		
120 min	25 ºC		

*When the duration of the process was 2 h, considering the temperature of the warmest season of the year in Puebla, the variability was 2.6ºC.

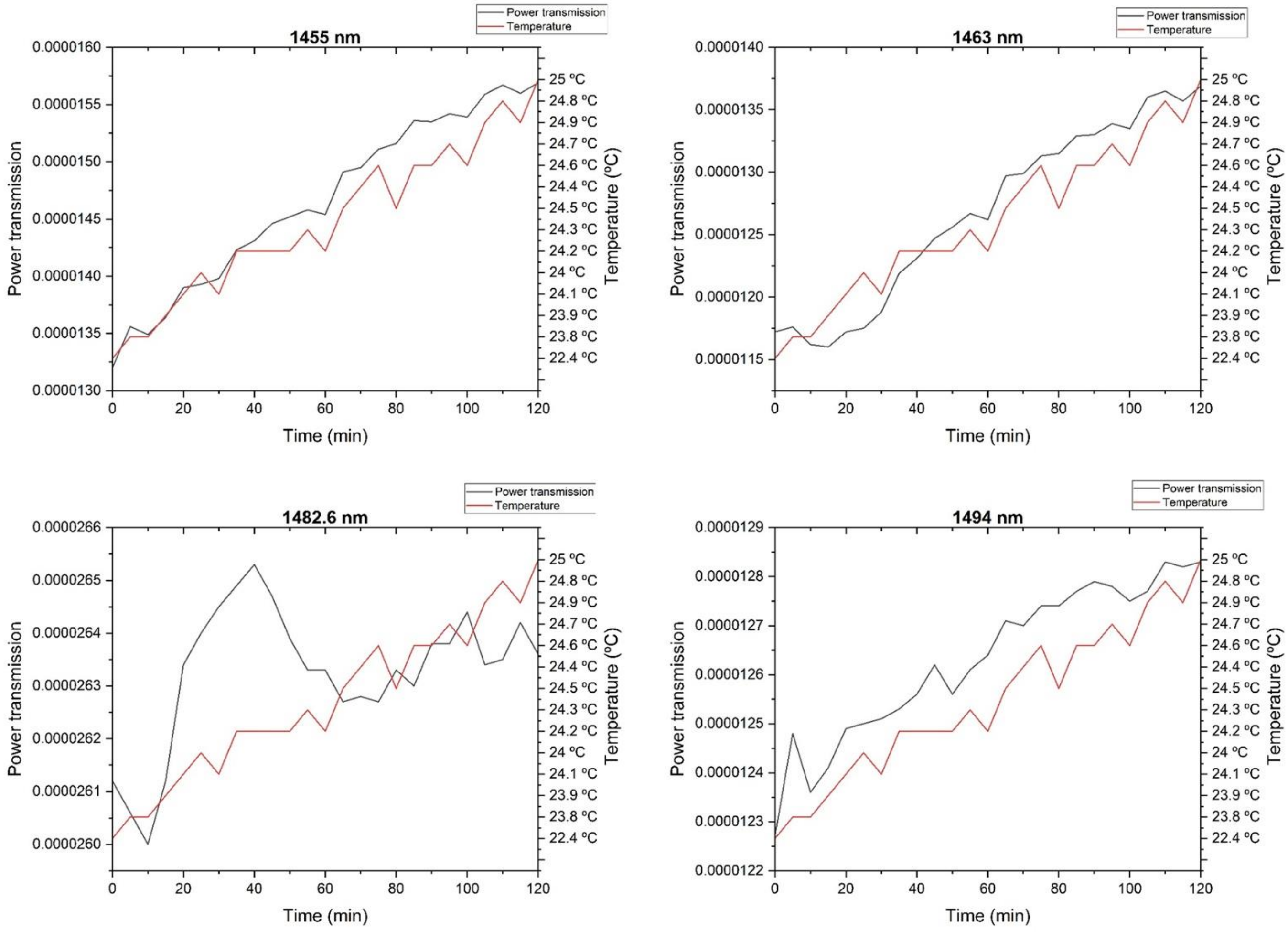

Figure S1.Monitoring of some wavelengths in the transmission spectrum during measurement by adding PBS.
