# Supplementary figures and images for: LPFG Biosensor for IL-6 Detection in Murine Serum Samples Associated with Ischemic Disease
Source: Sensors (Basel). 2026 May 2;26(9):2855. doi: 10.3390/s26092855 (PMC13165805; doi:10.3390/s26092855)

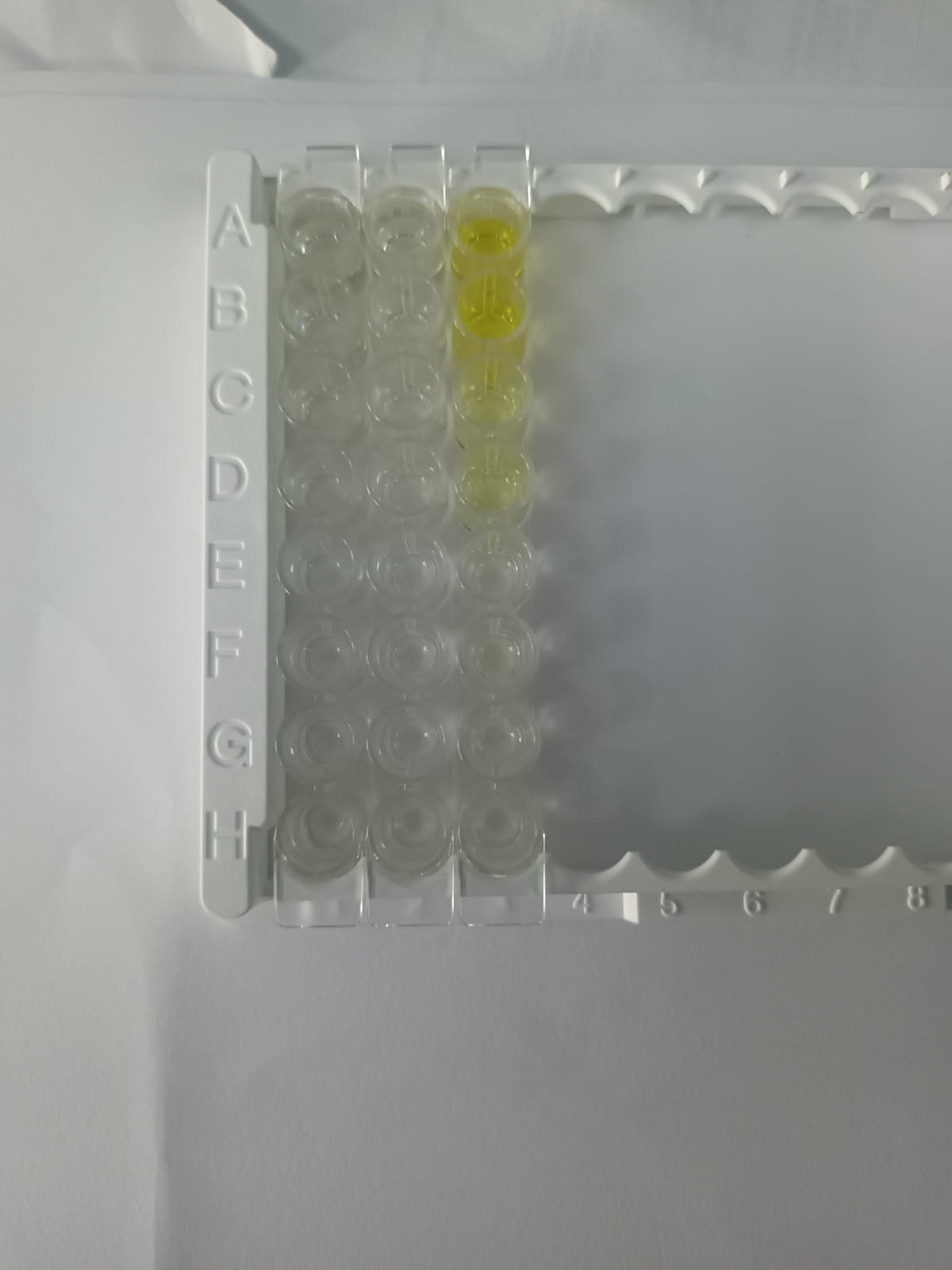

Supplement: Supplementary file 1 [file sensors-26-02855-s001.zip › Figure S2.jpg]
